# LifeSeeker: an interactive concept-based retrieval system for lifelog data

**DOI:** 10.1007/s11042-023-15317-w

**Published:** 2023-08-01

**Authors:** Thao-Nhu Nguyen, Tu-Khiem Le, Van-Tu Ninh, Annalina Caputo, Graham Healy, Sinéad Smyth, Minh-Triet Tran, Nguyen Thanh Binh

**Affiliations:** 1 Dublin City University, Dublin, Ireland; 2 University of Science, Vietnam National University Ho Chi Minh city, Ho Chi Minh, Vietnam

**Keywords:** Lifelog, Interactive retrieval system, Lifelog search challenge

## Abstract

Lifelogging was introduced as the process of passively capturing personal daily events via wearable devices. It ultimately creates a visual diary encoding every aspect of one’s life with the aim of future sharing or recollecting. In this paper, we present LifeSeeker, a lifelog image retrieval system participating in the Lifelog Search Challenge (LSC) for 3 years, since 2019. Our objective is to support users to seek specific life moments using a combination of textual descriptions, spatial relationships, location information, and image similarities. In addition to the LSC challenge results, a further experiment was conducted in order to evaluate the power retrieval of our system on both expert and novice users. This experiment informed us about the effectiveness of the user’s interaction with the system when involving non-experts.

## Introduction

In the past few decades, there has been an increasing interest in lifelogging applications thanks to the availability of low-cost and power-efficient wearable sensors and mobile devices recording multi-modal data including GPS, photos (egocentric photos), and biometrics data (heart rate, skin conductance response, and skin temperature). This has led to the creation of huge personal data archives capturing multiple aspects of a person’s life during a day for a long period of time [12], known as lifelog. Many research challenges, with related tasks and research studies, have been conducted to explore the potential applications of using lifelog data, such as Activities of Daily Living Understanding [5], Solve My Life Puzzle [6], Sports Performance Lifelog [21], and Lifelog Moment Retrieval [5, 6, 21] at the ImageCLEF conference. Among those tasks, the Lifelog Moment Retrieval (LMRT), which aims to develop a novel interactive/automatic retrieval system to search for specific moments of a person’s life, has gained much attention in the research community due to its potential development into a memory support technology [12]. Indeed, the LMRT task has been organised in different research challenges such as NTCIR Lifelog [7, 8], ImageCLEF Lifelog [5, 6, 21], and Lifelog Search Challenge (LSC) [9, 10] in different evaluation modes (online/offline) and with appropriate evaluation metrics.

Exploiting this large archive of egocentric images provided by the LSC’s organisers, we developed LifeSeeker, an interactive concept-based lifelog retrieval system in order to resolve the LMRT task in this challenge. LifeSeeker’s retrieval engine supports users with two search modalities including keyword-based search and visual similarity search. The keyword-based search retrieves relevant images by matching the input textual description with indexed images and metadata. Meanwhile, the visual similarity search uses the numeric vector representations of images to search for similar content. Furthermore, the User Interface is designed with a focus on ease of use aiming to maximize the user experience for all users.

The remainder of this paper is structured as follows. Section 2 provides an overview of the research related to image retrieval as well as several systems participating in LSC’21. Section 3 provides an overview of the task in the annual Lifelog Search Challenge with a brief description of the released dataset. Section 4 describes LifeSeeker’s system architecture, user interface and interaction. We discuss the detail of the search engine in Section 6, followed by outlining the system performance of LifeSeeker in LSC’21 in Section 7. Section 8 indicates how the experiment between novice and expert users is conducted in order to investigate the system’s performance. Finally, the conclusion of this work is drawn in Section 9.

## Related works

To address the problem of locating the desired life event in the LSC competition, various research teams attempted to build real-time interactive systems based on visual concepts while others exploited embedding models to bridge the semantic gap between text and image. Among those engines that leverage low-level visual features (such as objects, color, text, ...), Duane et al. achieved the best place of the first LSC in 2018 by introducing a user interface in Virtual Reality (VR) space [11]. Users were supported to interact and browse the immersive lifelog collections with a 360-degree view display. Myscéal [27] has been the state-of-the-art in the LSC for 2 consecutive years (2020, 2021), as it obtained the highest score within the shortest time. The authors’ key idea is to transform lifelogging images into a collection of visual annotations prior to matching them with the input keywords. Moreover, they assist users by not only implementing query expansion but also expanding the information related to location and text. Vitrivr [13], Vitrivr-VR [26], LifeGraph [23] and SOMHunter [18] are all participants in the Video Browser Showdown that adapted their video retrieval systems to the LSC challenge. In particular, Vitrivr [13] facilitates the browsing process by providing multiple search modalities via keywords, sketches, and audio. Additionally, image stabilization is applied as a pre-processing technique in order to enhance the quality of egocentric images. Vitrivr-VR [26], designed their system’s user interface in the VR space. The interactive retrieval process is eased with several VR-related functionalities such as an interactive map for spatial query formulation, sequence image view for spatial search, and cylindrical results view for result exploration. LifeGraph [23] takes a different stance by looking at the internal relations of the data collected from multiple modalities, which are then connected into the large static knowledge databases, ”Classification of Everyday Living” (COEL) and Wikidata, in order to provide more context for understanding the query. Beside the keyword-based search option, SomHunter [18] uses the weighted self-organising maps (SOM) to offer a wide exploration of the result. Their new version for LSC’21 is enhanced by integrating an embedding model as an extra search engine in order to enrich the contextual understanding. Memento [1] was introduced as a semantic-based retrieval engine that exploits the high-level visual embedding features to resolve the retrieval tasks. Instead of using keywords associated with the content, the developers represent both image and text queries as embedding feature vectors in the same latent space using an OpenAI-CLIP model [22]. This reduces the semantic gap of the visual-and-textual relation. Having a similar encoding model as Memento, Voxento [2] utilises voice control as their main modality to navigate the tool, which supports retrieval by providing users with a list of voice commands and interactions.

## The Lifelog search challenge

**Fig. 1:**
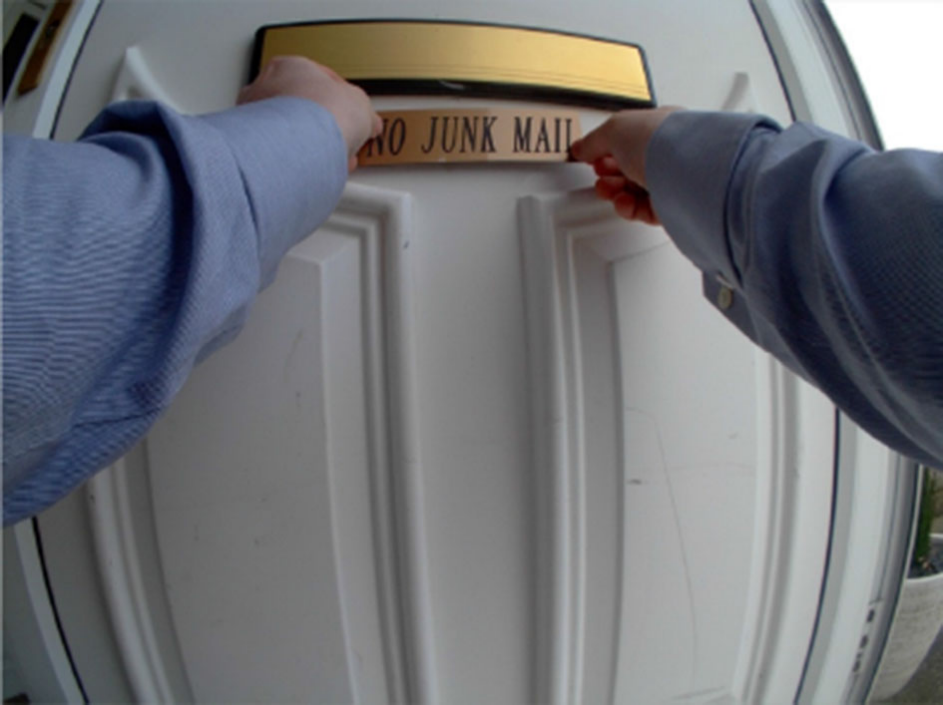
An example of search target image

As part of the ACM International Conference on Multimedia Retrieval (ICMR), Lifelog Search Challenge (LSC) is an annual lifelog retrieval competition first organised in 2018. From a given description, participants will handle the scenario of identifying one or more specific images related to the lifelogger’s activities within a given time constraint. An example of a query from LSC’21 is ”*I was getting too much junk mail so I put a sign on my door asking for no more junk mail. I remember I was wearing a blue shirt with cufflinks. It was in 2015 on the same day that I took a flight somewhere.*”. Images from the lifelogger’s log are considered relevant if they satisfy all clues from the given query (as shown in Fig. 1), otherwise, they are deemed as incorrect answers.

The LSC’21 dataset [9] is a collection gathered from NTCIR challenges, which contains multimodal data of one active lifelogger during the period of 114 days. The dataset was collected from multiple wearable devices including cameras, smartphones, and sensors. Particularly, a total of 183,299 images in 1024 × 768 resolution were captured by wearable cameras. Those egocentric images are fully-anonymised, i.e. human faces are blurred and sensitive texts are censored. Corresponding metadata, including date, time, and continuous location collected from the cameras were also provided by the organisers alongside images. In addition, we further extract annotations related to objects, textual information (OCRs), location, visual attributes, and categories which will be described in detail in Section 6.

## System architecture overview

Similar to most conventional retrieval systems, LifeSeeker [15], first released in LSC’19, is designed as a concept-based searching tool that relies on the analysis of both visual and non-visual content. Its original objectives were to provide users with the desired life moments of the lifelogger given a piece of textual information as a query, but also to improve user experience through a transparent and intuitive user interface. It is worth noting that the moment in this context is simply the single lifelog image that is captured by the lifelog camera at a certain time point in daily life.

**Fig. 2:**
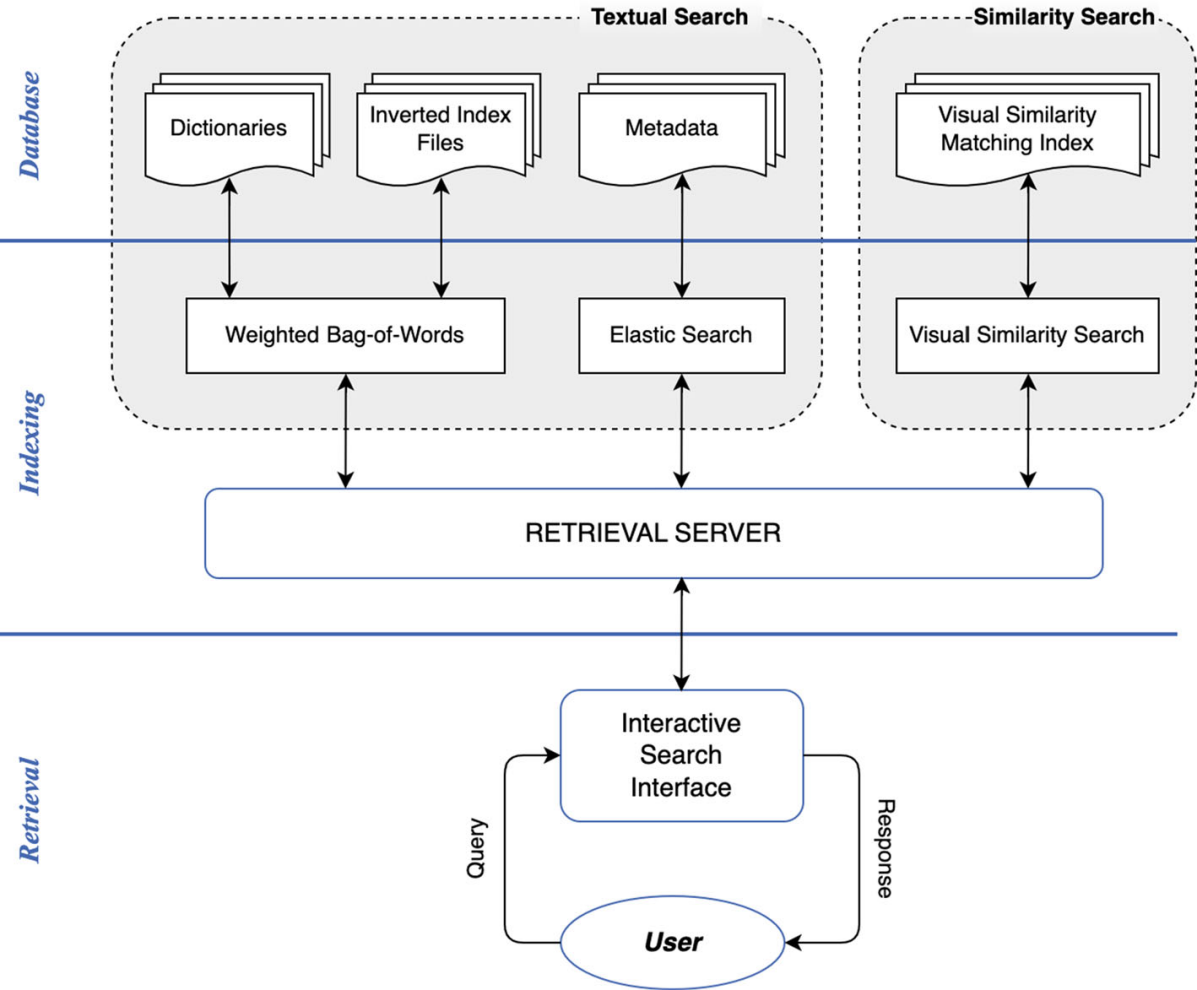
The System Architecture of LifeSeeker

Figure 2 illustrates the architecture of LifeSeeker, which consists of three components: a database, a retrieval engine, and an interactive search interface. The database component is responsible for storing the metadata and indexing embedded features extracted from the lifelog data while the retrieval engine utilises this information stored in the database to perform different forms of retrieval efficiently. The interactive search interface facilitates the interaction between the user and the system to perform search tasks. Compared to previous versions of LifeSeeker, the retrieval engine of the latest LifeSeeker system was enhanced by adding a Bag-of-Words model with visual concept augmentation. Its user interface (UI) was improved by enhancing the retrieval results’ display method [16]. The visual graphs were also implemented for both querying and filtering purposes to facilitate the browsing process. In the remainder of this paper, we will mainly concentrate on the latest version of LifeSeeker [20].

The database contains four different types of indexed metadata which are used in three different retrieval methods. In detail, the **Textual Search** component relies on three dictionaries (metadata-concepts, location, time) and an inverted-index file that maps the moment id (or the lifelog image id) in the format of YYYYmmdd_HHMMSS_000 where Y, m, d, H, M, S is the year, month, day, hour, minute, and second of the moment respectively; with its corresponding dictionary terms. On the other hand, the Elastic Search engine^1^ is used to index and retrieve both the metadata provided by the organisers combined with other metadata extracted from the collection. These include place categories and place attributes extracted from PlacesCNN [29], visual object concepts extracted from YOLOv4 [28] pre-trained on COCO dataset [17] and Bottom-up Attention Model [3] pre-trained on Visual Genome dataset [14], and text extraction data (OCR) with other visual concepts extracted using Google Vision API^2^ and Microsoft Vision API^3^ respectively. Detailed descriptions of the three dictionaries and the Weighted Bag-of-Words retrieval method are provided in Section 6.2.1; while the structure of the indexed metadata file created for the Elastic Search engine is detailed in Section 6.1. For the **Similarity Search** component, the ranked list of visually similar images of a specific-target photo obtained from the Visual-Similarity Search algorithm (Section 6.2.3) is stored in the MongoDB database to boost the speed of the visual similarity search.

The LifeSeeker’s retrieval server is developed using the Django framework^4^ which plays the role of a middleware supporting the communication between the client-side requests (user interface and interaction) and different retrieval modules. In general, the core retrieval engine consists of two components: **Textual Search** and **Similarity Search**. In our system, the **Similarity Search** component contains only one module which is the Visual-Similarity Search described thoroughly in Section 6.2.3. This module takes an image as input and returns a ranked list of photos that have similar visual patterns (e.g. objects’ edges/angles/orientations) to the input. The **Textual Search** component of LifeSeeker consists of two retrieval modules: Weighted Bag-of-Words and Elastic Search, which are also the two core retrieval modes of the system. The Weighted Bag-of-Words module is a free-text search that takes the description of the life-moment as input for retrieval; while the Elastic Search requires the user to input query terms, which are manually parsed from the description, following a pre-defined syntax. In short, the decision of which component and which module to use for retrieval is determined by the retrieval server based on the input type (full sentence, query terms in a pre-defined syntax, or images). Our design of the retrieval server aims to support the simplicity of the user interface and user interaction, which reduces the learning curve of using the system efficiently for novice users while preserving the efficiency of the system for expert users.

The interactive search interface of the LifeSeeker is a web-based application developed using ReactJS framework.^5^ The main components of the LifeSeeker’s user interface (UI) are the free-text search box, the vertically-scrollable panel displaying the retrieval results, and the detailed box showing related contents of the selected image including visually similar moments, preceding moments and successive ones. The user provides the query to the system using the search box by entering either query terms following a pre-defined syntax (described in Section 6.2.2) or a full sentence describing the desired life moment. Matched lifelog images are then displayed on the vertically-scrollable panel for further browsing or scanning interaction. Detailed descriptions of the user interface and user interaction are presented in Section 5.

## User interface and user interaction

### User interface

**Fig. 3:**
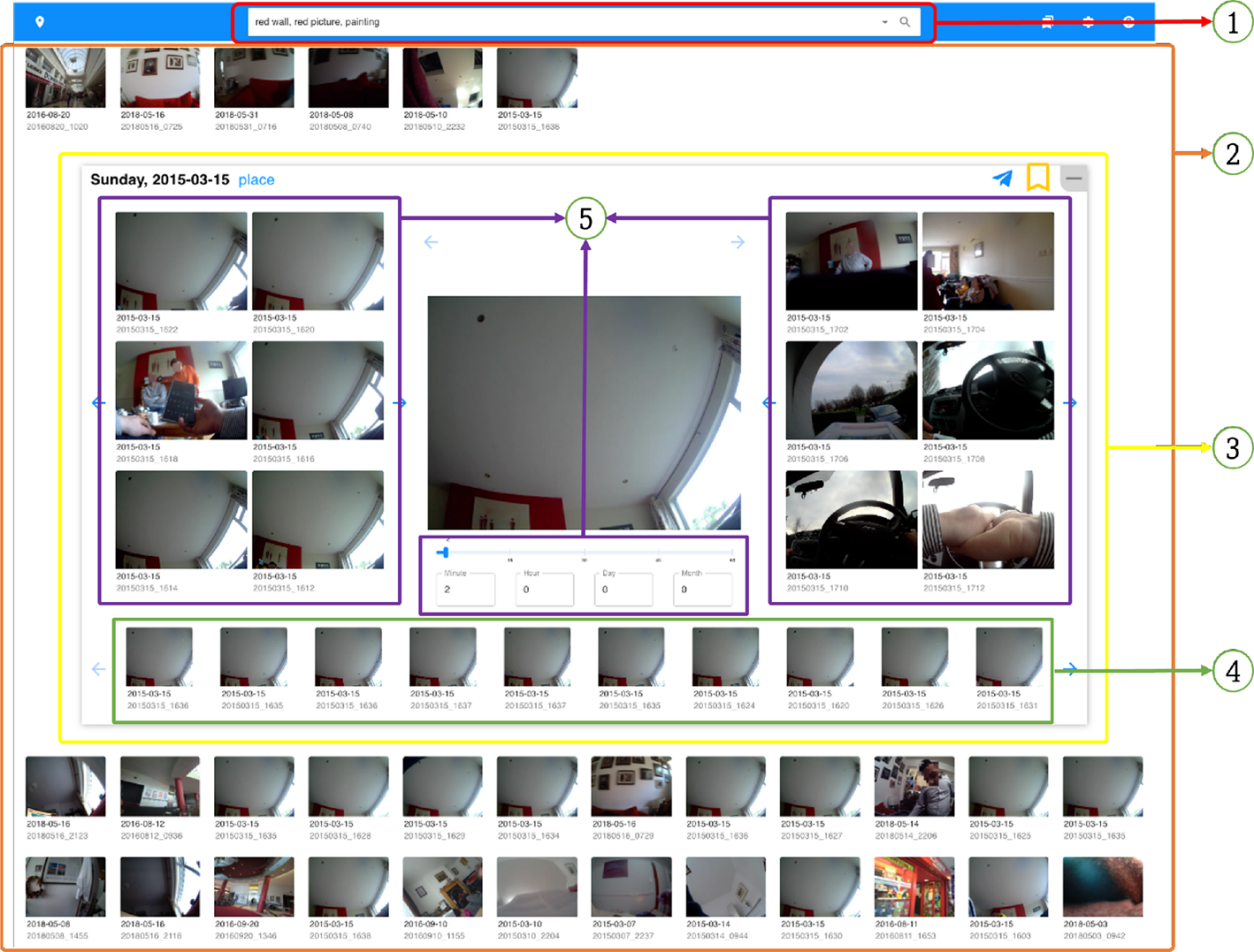
The Interactive User Interface of the LifeSeeker Retrieval System

The interactive user interface of LifeSeeker is composed of three main components (Fig. 3), which are the free-text search box **(1)**, the vertically-scrollable panel displaying a ranked list of retrieved moments **(2)**, and the moment-detail box **(3)**. The vertically-scrollable panel (**2**) shows a ranked list of retrieved moments obtained from the query submitted in the free-text search box (**1**). Each item in the panel is a square box displaying the lifelog image with minute id (an example of the minute id is shown in the Listing 1) and captured date with format YYYYmmdd_HHMM and YYYY-mm-dd respectively where Y, m, d, H, and M denotes the year, month, day, hour, and minute correspondingly. The vertically-scrollable panel and its items are designed for optimal moment-scanning and browsing on a 27-inch monitor (367.69 mm × 612.49 mm). Specifically, on a 27-inch monitor, there are at most five rows of images in normal-screen mode and six rows in full-screen mode. Each row of the panel consists of at most 12 items showing the lifelog moment with its date and time. It is worth noting that this optimization is specifically designed for the Lifelog Search Challenge to reduce the overhead time to find the correct moments to submit by scrolling up and down. According to our experience in previous Lifelog Search Challenges, viewing as many top results as possible without scrolling can result in a big gap in the score and the rank of top-performance systems in the competition.

For each image shown on the vertically-scrollable panel, we decide to show its date and time of the moment as it is considered to be one of the most important pieces of information of a lifelog moment that cannot be recognized visually from the image. For the moment-detail box (**3**), the lifelog image of the selected moment is shown in the middle of the box.

Apart from the lifelog image of the selected moment located in the middle of the moment-detail box (**3**), there are two other essential components; these are the horizontal panel displaying visually-similar images and the temporal browsing panel in component (**4**) and (**5**) respectively. The horizontal panel (**4**) displays at most 10 images, which are visually similar to the selected moment in terms of objects’ edges/angles/orientation, on the 27-inch monitor. The temporal browsing panel (**5**) consists of two horizontally-scrollable panels on both the left and the right of the lifelog image in the middle of the moment-detail box (**3**) showing a sequence of moments that happen before (left) and after (right) the selected moment. In addition, a before-and-after time-range controller is placed under the selected moment. This time-range controller is used to adjust the temporal range the user would like to explore from the selected moment. By adjusting the time delta, images before and after the target photo can be adjusted to be temporally nearby or further apart. It is our conjecture that the target memory is usually retrieved by connecting the previous memories, which form a path that leads to the piece of memory during the recalling process. The query “Eating fishcakes, bread and salad after preparing my presentation in PowerPoint” would be a good example to clarify our conjecture. Querying for the moment when the lifelogger had fishcakes, bread, and salad is not enough to uniquely identify the desired moment among a thousand of the same ones without considering the temporal-activity information. Therefore, the temporal browsing panel (**5**) is an essential part of the user interface when dealing with temporal queries.

### User interaction

The full flow of user interactions can be illustrated via four steps:

1. The user inputs the query into the search box (**1**). The query can be in the form of a full sentence describing the moment or in the form of a sequence of terms following the syntax (2). The search box (**1**) also supports the term auto-completion to facilitate the user inputting the query.
2. The user can either scan or browse the ranked list of relevant images displayed on the vertical-scrollable panel (**2**).
3. Any moment for which the user wants to investigate if it is the answer to the query, the user has two options to browse it further; these are left-clicking on the image to open the moment-detailed box (**3**) or hovering on the image while pressing the X key to enlarge the image.
4. In case the user opens the moment-detailed box (**3**) for further browsing, the user can use the temporal browsing panel (**5**) to view the previous/after moments of the selected one by horizontal scrolling the panel as well as adjusting the time delta to view the temporal nearby or further apart moments.

These four steps are performed repeatedly during the search process. It is worth noting that the search box is also capable of performing a filter search by inputting the query terms following the syntax (2).

## Search engine

This section is dedicated to detailing all components of the search engine that powers LifeSeeker. As LifeSeeker was specifically designed to address the LSC challenge (which aims to retrieve lifelog moments based on cues given by a lifelogger), its search engine takes as input a text-based query and returns a list of moments (represented by images) ranked by the descending order of their relevance to the query. To achieve this, LifeSeeker is equipped with two main modules: (1) an indexing module that processes the input data (images, biometrics data, metadata) from the dataset and transforms them into a searchable representation; (2) a retrieval module which takes an input query and matches it with the data previously processed by the indexing module to return the relevant moments.

### Indexing

**Listing 1:**
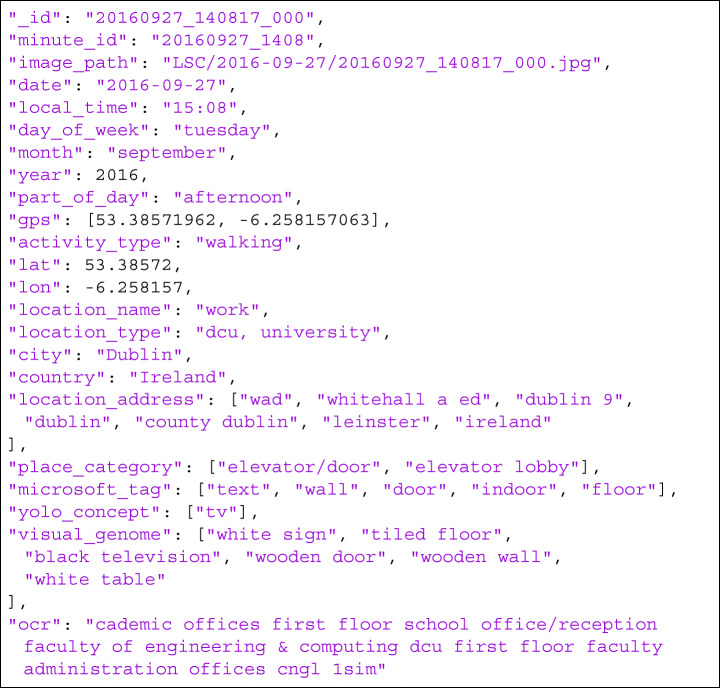
A sample metadata for a lifelog moment generated by the Indexing module

Since the lifelog dataset is constructed by gathering data from multi-modal sensors (i.e. wearable cameras, biometric devices, GPS, phones, computers), the **Indexing** module requires various sub-modules, each responsible for processing one modality of the lifelog data. Inspired by the lifelog data analysis from NTCIR-14 Lifelog-3 task, we categorize the lifelog data into the followings:

1. **Time**: This is one of the most important pieces of information that helps to narrow the search space greatly. For example, knowing when (morning, afternoon, evening) the moment happened can filter out nearly two-thirds of the original amount of images. Section 6.1.1 describes the process of indexing the time data in more detail.
2. **Location**: Location can be viewed as a summary of a lifelogger in terms of where they were on a daily basis, which might imply the sequence of activities that the lifelogger does throughout the day. It is also useful for adding more context to the query generation process to find more relevant moments (i.e. if finding moments that the lifelogger was eating a sushi platter, the user can add ”Asian restaurant” as part of the LifeSeeker input query to obtain more accurate results). The indexing pipeline for location data can be found in Section 6.1.2.
3. **Visual concepts**: Images captured from the wearable camera are information-rich, as moments are illustrated in detail (i.e. how the surroundings look like, who appears in that moment, and which objects are seen). However, computers cannot perceive images as humans do. Therefore, in Section 6.1.3, we outlined several adopted approaches to convert images into a list of visual concepts that a machine can read and process.
4. **Other metadata**: Apart from the aforementioned data sources, there are other modalities provided in the dataset as listed below. However, this metadata can be indexed instantly into the search engine without further processing.
  - **Activity**: The activity data contains two categories: walking and transport.
  - **Biometrics**: The biometrics data that we use in our search engine includes heart rate and calories.

#### Time

When referring to time, we have different ways to describe it. For instance, “*September 27, 2016 at 15:08*” can be referred to as “*2016/09/27 at 15:08*”, “*Tuesday afternoon in September 2016*”, or “*September 2016, after 3 pm*”. Therefore, to handle input queries containing variable time formats, these different variations need to be indexed in the search engine in advance. We note that the local time gives a more intuitive view into a day in the lifelogger’s data, compared to the standard UTC time collected from wearable devices, especially when the lifelogger was traveling to another country in another hemisphere. Hence, we aligned the current time into the local timezone at the location where the lifelogger was at that time. Since lifelog data is organised on a one-minute basis, each image has a *minute_id* that we can process as follows:

- Date: The date of the image, in the YYYY-MM-DD format;
- Month: Name of the month (e.g. January, September, December);
- Year: The year in the YYYY format;
- Local Time: The time in the lifelogger’s local timezone in 24-hour format;
- Day of Week: One of the seven days of the week expressed in the lifelogger’s local time;
- Part of the Day: Whether it is *early morning* (04:00 to 07:59), *morning* (08:00 to 11:59), *afternoon* (12:00 to 16:59), *evening* (17:00 to 20:59), or *night* (21:00 to 03:59), based on the local time.

A sample of the generated time data is illustrated in Listing 1 in the fields *date*, *month*, *year*, *local_time*, *day_of_week*, and *part_of_day*.

#### Location

Another important attribute in every lifelogger’s life moments is the locations they have been. Knowing the correct location would give more meaningful information to expedite the search process. From the geographic coordinates collected from wearable devices, we identify the detailed address of the image using Geocoding API from Google Map Platform.^6^ Apart from the address, city and country also play a crucial role in the filtering process, especially for locations outside Ireland (where the lifelogger is based). Moreover, we also cluster the locations into 32 pre-defined place categories. Each image has information related to the location of the lifelogger at that moment as follows:

- Latitude: Angular coordinate specifies the north-south position of the image on the surface of the earth;
- Longitude: Angular coordinate specifies the east-west position of the image on the surface of the earth;
- Location’s name: Semantic name of the location (i.e. Dublin Airport, DCU, ...);
- Location’s type: One of the 32 predefined categories in Table 3;
- Location’s address: Detailed address associated with the lifelogger’s location;
- City: Name of the city associated with the lifelogger’s location;
- Country: Name of the country associated with the lifelogger’s location.

#### Visual concepts

**Fig. 4:**
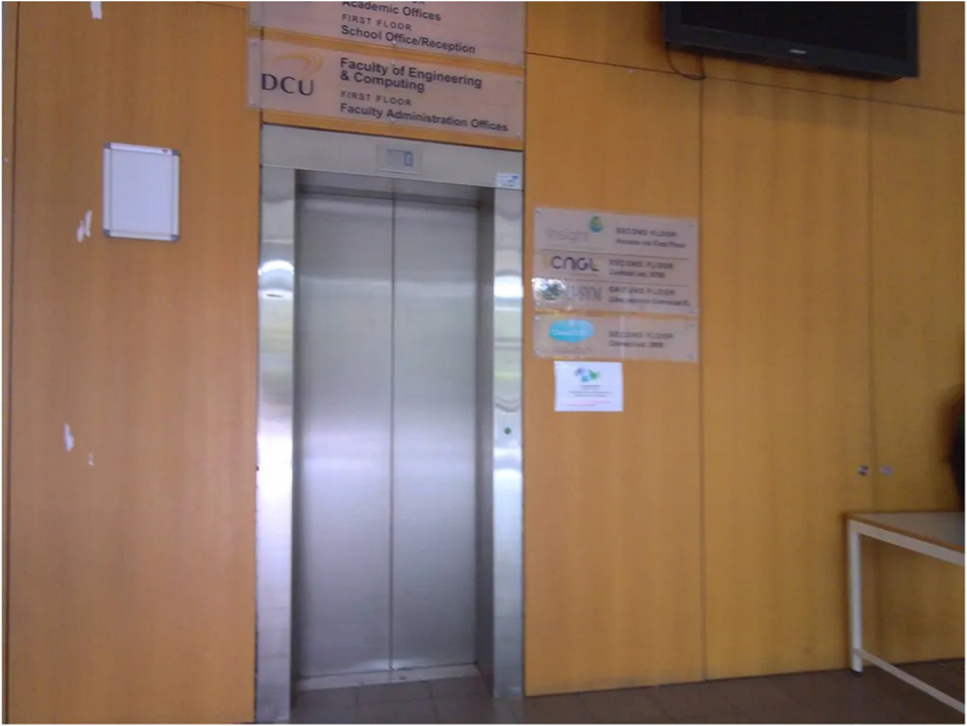
The corresponding image as described by the concepts in Listing 1

**Text recognition**: Texts appearing in lifelog images can help to determine not only what the lifelogger might have seen, but also the context of the associated life moment. Therefore, to convert texts in lifelog images into visual concepts, we employed the OCR tool from Google Vision API to detect and recognise text content. The extracted texts were then aggregated into a single string (as shown in Listing 1 in the *ocr* field) that can be indexed by the search engine in the latter stage.
**Object detection**: Object tagging is an essential component for most concept-based retrieval systems. Thus, visual concepts of lifelog images, obtained from object detection models, are always provided as part of the lifelog dataset in all collaborative research tasks and challenges in the lifelogging domain [10]. Besides the visual concepts shared by the lifelogger/task organisers, which were generated using Microsoft Vision API, we also considered other object detection models (e.g. YOLOv4 and Bottom-up Attention model) with the aim of tagging more objects from lifelog images. The YOLOv4, which was pre-trained on the COCO dataset, can detect 80 different categories of common objects in daily life. Meanwhile, the Bottom-up Attention model is able to detect 1600 object classes along with 400 associating attribute types (i.e. black pillar, wooden floor, red car, etc.) by using multi-GPU pre-training of Faster R-CNN with ResNet-101. This model not only increases the number of concepts by a significant amount but also enables the retrieval of concepts at a finer level of detail using their corresponding attributes. The fields *microsoft_tag*, *yolo_concept*, and *visual_genome* in Listing 1 illustrate a sample result of the visual concepts generated by Microsoft Vision API, YOLOv4 and Bottom-up attention model, respectively.
**Scene recognition**: In addition to text and object concepts, understanding of the surroundings also gives more insight into where the lifelogger was (i.e., waiting in a lobby, exercising outdoors, working in an office). To achieve this, we utilised the PlacesCNN [29] model pre-trained on the Places365 dataset, which classifies images into 365 place categories. For example, the lifelog moment displayed in Fig. 4 was recognised as ”*elevator/door*” and ”*elevator lobby*” as shown in the field *place_category* in Listing 1).

### Retrieval

#### Weighted bag-of-words

We implement a customized Bag-of-Words algorithm that serves both free-text search and filtering. Firstly, three dictionaries are generated from the pre-processed metadata that includes time, location, and visual concepts:

- **Time dictionary**: consists of the information of the month (from January to December), weekday (from Monday to Sunday), and part of the day (early morning, late morning, afternoon, etc.).
- **Location dictionary**: consists of semantic location names, countries, cities, and place categories obtained from PlacesCNN.
- **Visual-concept dictionary**: consists of multiple object labels extracted from deep-vision model pre-trained on MS-COCO and Visual Genomes dataset as well as the ones obtained from Microsoft Vision API.

The dictionaries are refined using the *nltk library*^7^ so that stop-words are removed. In addition, we also manually filter the dictionaries to remove meaningless terms as well as one-character terms and non-alphabetic characters. Unlike the traditional Bag-of-Words, in our algorithm, we do not consider inverse document frequency (IDF) weighting since occurrences of terms in the corpus are all considered to be equally important; the dictionary weight is used instead. This is because the IDF weighting would reduce the significance of some common terms which frequently appear in the lifelog annotation corpora such as week-of-day, part-of-day, and semantic location labels. The dictionary weight (*w*) is variable and changes to reflect the importance of each dictionary. In our case, Let *w*_time_, *w*_loc_, and *w*_vc_ be the weights of the time, location, and visual-concept dictionaries respectively; then we set *w*_time_ > *w*_loc_ > *w*_vc_. The time information is essential to identify a specific moment and filter the results. In addition, the location dictionary is considered more important than the visual concept one (*vc*), as it would be easier to navigate to the desired moment if the location is given in the query. These weights are combined into a vector and then are multiplied into the L2-norm term frequency vector of the query to amplify the time and location when computing cosine similarity between the query vector and the L2-norm term frequency vector of images in the archive to retrieve relevant images. To summarize this idea, we use the following formula for our re-defined cosine similarity computation between the weighted Bag-of-Words query vector and the ones in the archive. Let ***tf***_***q***_ and ***tf***_***i***_ be the L2-norm term-frequency vector of the query and the L2-norm term-frequency vector extracted from the annotation of the lifelog image ***i*** in the dataset respectively. The term weighting of the three dictionaries (time, location, and visual concepts) is represented by a vector ***w***. The score computed from our re-defined cosine similarity is shown in (1) where ⊙ denotes the pairwise multiplication operation between two vectors.

1 $$  \text{score} = \frac{(\boldsymbol{w} \odot \boldsymbol{tf_{q}}) \cdot \boldsymbol{tf_{i}}}{\|\boldsymbol{tf_{q}}\| \|\boldsymbol{tf_{i}}\|} $$

#### Elastic search

Elastic Search is another search mode that is used in LifeSeeker, first introduced in the second version [16]. Despite the general underlying mechanism of Elastic Search being similar to the Weighted Bag-of-Words approach (Section 6.2.1), Elastic Search provides not only speed and scalability to the search engine of LifeSeeker, but also many modules for indexing, querying, matching and filtering data. A query into Elastic Search can be constructed by combining one or more *query clauses*^8^ of various types, thus users can form very complex queries to define how Elastic Search retrieves data. Therefore, this search mode was intentionally integrated for expert users for competing in the LSC challenge.

In order to reduce the query analysis time and allow flexibility in controlling how each keyword should behave when retrieving lifelog moments (i.e., which should be used for matching images and which should be used for filtering purposes only), we introduced a syntax-based query mechanism as below:

2 $$  \texttt{<CONCEPTS> ; <LOCATION> ; <TIME>} $$

where each query part (<CONCEPTS>, <LOCATION> and <TIME>) corresponds to a category outlined in Section 6.1. A syntax-based query can be formed by specifying keywords in each part in Syntax 2. For instance, the following query is a valid input to LifeSeeker:

$$\texttt{ flower teddy bear ; bedroom home ; after 7pm on Monday}$$

The Searching process in Elastic Search mode was done by employing the *query string query*^9^ to match <CONCEPTS> and <LOCATION> keywords, while the *term query*^10^ and *range query* mechanisms were used to filter images using the given <TIME> keywords.

#### Visual similarity search

For visual-similarity search, we utilise the Bag-of-Visual-Words model to transform visual features into a vector representation for the K-Nearest Neighbors algorithm. In general, the algorithm of the Bag-of-Visual-Words model is similar to the traditional Bag-of-Words one used in textual information retrieval except for the creation of the dictionary, which is usually known as the visual codebook. Each item in the visual codebook is called the visual word instead. In the Bag-of-Visual-Words model, the visual codebook can be constructed using the K-Means Clustering approach that clusters the descriptors extracted from Scaled-Invariant-Feature-Transform (SIFT) [19], the Oriented FAST and Rotated BRIEF (ORB) [24], and Speeded Up Robust Features (SURF) [4]. It is worth noting that an image can have many descriptors, therefore, resulting in having many visual words. The choice of the parameter K in the K-Means Clustering algorithm determines the number of visual words in the visual codebook. In LifeSeeker, we use 256-dimensional descriptors of ORB features as inputs for the visual codebook generation process. Due to the huge number of descriptors in a large-scale dataset, we employ the Mini-batch K-Means Clustering described in [25] to reduce the computation cost while gaining asymptotic clustering results compared to the conventional K-Means Clustering approach. The Mini-batch K-Means Clustering is performed with 50 iterations. The value of K used in our case is 4096 as we consider this number of visual words is enough for a visual-similarity search. All the remaining steps including vector quantization and similarity computation are performed as in the traditional Bag-of-Words model. For computing similarities between images, the cosine distance function is employed instead of the Euclidean distance function.

## Benchmarking result in lifelog search challenge

LifeSeeker was benchmarked in the fourth annual Lifelog Search Challenge (LSC’21) along with 15 other retrieval systems. The ultimate goal of the challenge is to retrieve the relevant lifelog image that matches a given query as fast as possible. In addition, penalties are applied for wrong submissions. The challenge was conducted in an interactive manner, which means that there was one user using the system to perform the search and submit the image that they think best illustrated the query. For each task, the score [11] of one LSC participant retrieving the correct answer at a time *t* is officially calculated as follows:

3 $$  S_{i} = \max \left( 0, M + \frac{D-t}{D} \* (100-M) - W*10 \right) $$

where *M* refers to the minimum score earned, *D* denotes the query’s duration and *W* represents the number of wrong submissions for each query. Specific to this case, *M* and *D* are set to 50 and 300, respectively. As can be seen from the formula above, the score is linearly decreased until the minimum score (50) within the 300-second period. Then the final score is taken by subtracting each negative submission by 10 points. A participant gets a zero score when the time for the query is over (300 seconds have passed) and a positive answer was not found.

LifeSeeker was the third best performing system in the challenge which achieved a total score of 1556.02 (Table 1). As shown in the table, LifeSeeker had the most queries solved among the top-5 systems (solved 20 out of 23 queries). Along with the score, we would also evaluate our system’s performance through other measurements such as precision and recall. Precision is the number of correct images out of total submissions, while recall refers to the number of queries solved over total queries. LifeSeeker got the highest recall score of 0.87. This benchmarking result indicates that our proposed system is capable of retrieving the desired information up to 87% of the time (4.35% more than that of the second-highest recall system). In regard to the precision, LifeSeeker achieved 0.77, which is 8.8% lower than Voxento [2] (system with the highest precision). The direct cause of our lower precision was the number of incorrect submissions that the system user made during the challenge.

**Table 1:** Statistics of the top-5 teams in LSC’21

Team name	Queries solved	Total score	Precision	Recall
Myscéal [27]	19	**1604.3065**	0.8261	0.8261
SomHunter [18]	19	1566.3177	0.6786	0.8261
Voxento [2]	18	1466.8732	**0.8571**	0.7826
Memento [1]	16	1238.4937	0.5926	0.6957
LifeSeeker [20]	**20**	1556.0233	0.7692	**0.8696**

**Fig. 5:**
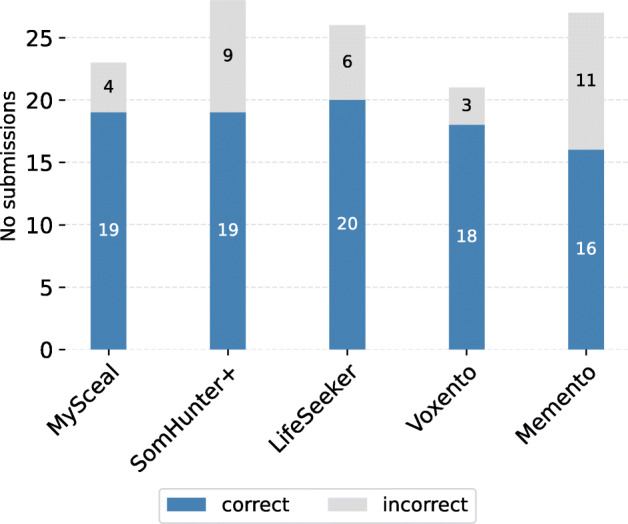
The number of correct and incorrect submissions of the top-5 teams across all queries in LSC’21

As illustrated in Fig. 5, LifeSeeker had 6 incorrect submissions, which not only influence the precision, but also the total score (penalty counts towards the number of wrong submissions). This explains why the system came third in the challenge, though it solved more queries compared to other retrieval systems in LSC’21.

## Experiments and evaluations

Apart from the benchmarking result obtained from the LSC’21 challenge, we also conducted an experiment to further evaluate the performance of the system when being used by a person who has no prior knowledge about lifelogging and lifelog retrieval systems, whom we referred to as **novice** users. The rationale behinds this user study is to seek improvements for the system participating in future LSCs, as novice session has been part of the LSC since its beginning (except LSC’20 and LSC’21 due to the pandemic which made the challenge went virtual). LifeSeeker was bench-marked with both expert and novice sessions in LSC’19, but only the expert session in LSC’20 and LSC’21. Therefore, most of the upgrades introduced to the latest system were to enhance the experts’ performance in the challenge. Through this experiment, we want examine how novice users perform compared to expert ones so that we could gain more insights into which functions of the system should be kept, as well as what features we should introduce to the future versions of LifeSeeker to increase its performance when being used by both expert and novice users.

In order to maintain the consistency of the evaluation metrics used to generate benchmarking results (i.e. used in the actual challenge), we simulated the LSC competition protocol by adopting the queries from the challenge and replicating an evaluation platform similar to *DRES*.^11^ Experimental participants were recruited and guided to use the system to solve the queries as if they were participating in a real LSC.

### Experimental protocol

#### Participants

There were 2 groups of users participating in the experiment: Novices and Expert. ***Novices*** are those who have little to no knowledge about either the lifelogger or the system in general, while the ***Expert*** (literally the system developer) is familiar with the functions of the system in overall and a part of the lifelog data. With regard to the novice users, a total of 4 volunteers (3 postgraduate students and 1 researcher denoted by “*User 1*”, “*User 2*”, “*User 3*” and “*User 4*”) were recruited without any specific technical requirements. The expert involved in this experiment was not part of the experiment setup or the query choice process; this was to guarantee the fairness of the experiment.

#### Experiment design

Prior to the experiment, we introduced the volunteers to how the system operates, how to navigate the search tool, and how to get useful information from the result display. Afterward, they were provided some test queries to get used to the system. There are 10 queries collected from the query set given by the LSC organisers with a full description shown in Appendix Appendix. Every initial information is provided as a piece of text, which is then followed by 5 further hints each displayed at intervals of 30 seconds. Consequently, the maximum search time allowed for one query is 3 minutes, leading to a duration of 1 hour per user. There is no limitation on the number of submissions, either relevant or irrelevant. Users are able to submit as many images as they like until the correct one is found. However, as explained in Section 7, wrong attempts do have a negative impact on the overall results in terms of score, precision, and recall, and also result in a 10-point penalty on the official total score.

For further analysis purposes, the statistics related to searching time, score, and correctness have been recorded at the end of each query. Particularly, solving time and score were calculated for the first correct answer only. The score *S* of the positive answer at time-step *t* was calculated based on the same scoring scheme used in the LSC (3) with *M* and *D* of 50 and 180, respectively.

### Experimental results

Table 2 reports the performances of the novice users benchmarked against the expert. In general, the expert user achieves a better score in total, which is about 180 points higher than the best score of newcomers (346.95). Having insights into the dataset undeniably is one of the expert’s advantages in some cases. As in Q2 and Q8: while the novices struggle to locate the target, the expert puts less effort into the search process. This is highlighted by the high difference in scores (up to 90 points in some cases) that are registered in those events. Queries related to daily activities such as drinking beer during BBQ (Q6) or eating at home (Q9) seem to be challenging for all users since there are many similar events happening at that time and the given description is not detailed enough to distinguish between them. Surprisingly, the very last query (Q10) is solved by all novices except the expert. That query is a tricky one with confusing information about the car.

**Table 2:** Details of score per user across all queries

Query ID	User 1	User 2	User 3	User 4	Expert
Q1	66.94	65.83	43.61	50.28	**71.26**
Q2	0.00	0.00	0.00	0.00	**71.67**
Q3	58.89	56.39	46.67	**68.34**	50.56
Q4	73.61	77.78	15.00	66.94	**96.11**
Q5	71.67	0.00	86.94	0.00	**90.85**
Q6	0.00	0.00	0.00	0.00	0.00
Q7	0.00	60.28	0.00	0.00	**74.60**
Q8	0.00	0.00	0.00	0.00	**68.75**
Q9	0.00	0.00	0.00	0.00	0.00
Q10	58.61	**86.67**	57.50	74.44	0.00
**total**	329.72	346.95	249.72	260.00	**523.80**

The highest score of each query is highlighted

**Fig. 6:**
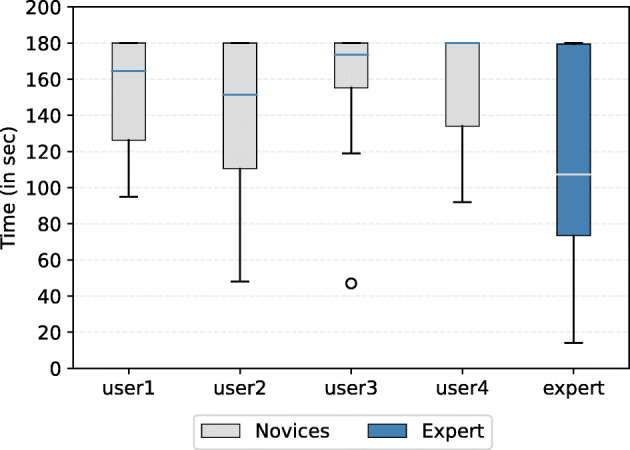
The distribution of search time per user across all queries. *Novices* denotes the group of newcomers, while *Expert* refers to the system designer

**Fig. 7:**
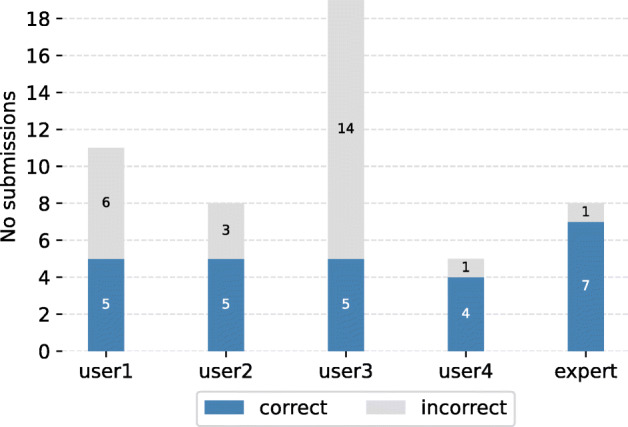
The number of correct and incorrect submissions per user across all queries

We note that by comparing the solving time distribution (Fig. 6), we gain more insights into how efficiently each user performed. Since any incorrect answer will be penalized by an amount of 10 points, it is important to highlight the number of both relevant and irrelevant answers. Apparently, the newcomers tend to have more wrong answers compared to the expert, as can be seen from Fig. 7. Despite solving the same number of queries as user 1 and user 2, user 3 has the highest number of negative answers (13 answers) which results in a huge gap between their scores (more than 60 points).

**Fig. 8:**
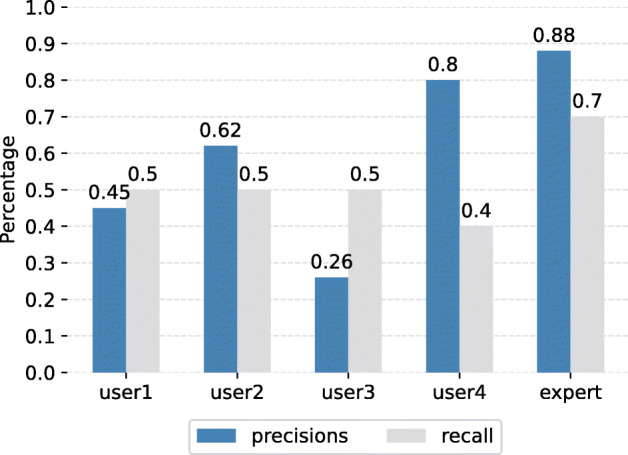
Precision and Recall per user across all queries

We also take recall and precision (illustrated in Fig. 8) into consideration since they are key measurements to evaluate the system’s efficiency. Those values are calculated using the ranked images and a list of relevant images according to the query number. The precision and recall are calculated by the same formula as mentioned in Section 7. Consequently, with the smallest number of both correct and wrong submissions, not to mention the shortest solving time, the expert user is undeniably the one with the highest score in both measures. Although 6 out of 10 queries were solved (the highest among the newcomers’ group), user 3 obtains a precision of just 0.3 due to the high number of incorrect answers (Fig. 7).

In summary, the experimental results showed that the expert user outperformed the novice ones with the highest score, highest precision, and recall within the shortest amount of time. The concept of LifeSeeker is a keyword-based search tool that relies on the tagged visual keyword collection. When using a concept not tagged in the image’s metadata, the search engine fails to retrieve relevant images although the concept is a synonym, and hence correct. That could explain why the expert, who has a wealthier knowledge of terms, has advantages in the search process. Moreover, the system developer is more familiar to interact with the system as compared to the newcomers could lead to a shorter search time. To bridge the gap between the performance of the expert and novices, it is necessary to consider improving the system by leveraging more information such as semantic meaning between objects or additional metadata, instead of relying on visual concepts only.

## Conclusion

In this paper, we present the third version of LifeSeeker, a concept-based lifelog retrieval system, which was first introduced in 2019. LifeSeeker consists of an interactive easy-to-use user interface and a fast scalable search engine. The interface enhances lifelog moment browsing through various means of interaction with retrieval results such as moments’ metadata details, adaptive temporal display, and visually-similar moments exploration. In regard to the search engine, it is responsible for indexing lifelog images’ metadata into databases and analysing user input free-text query to match the query’s terms with the previously indexed images’ metadata to return relevant results. The interface’s design choice and search engine implementation details were explicitly outlined with a clear rationale behind each decision in Section 4.

LifeSeeker has been an active participant in the LSC competition for several years; through this period of time, we have benchmarked our system against state-of-the-art competitors gaining insights into the lifelogging search task. In the latest LSC (2021), LifeSeeker achieved competitive results being the top-3 best-performing system in overall score and the top-1 system with the highest number of queries solved (highest recall score). In addition to the benchmarking results at LSC, we also conducted a user study to evaluate the usability and performance of the system when being used by a novice user who does not know about lifelog and retrieval systems. The experimental result showed that there is a gap in the novices’ performance compared to that of the experts (the system’s developer). From the analyses from our experiment, we could so far gain an initial insight that one of the reasons of the huge gap between the expert and novice users is the concept-driven characteristic of LifeSeeker which limits the number of terms that one can use in their query.

## Appendix A: Queries used in the experiments

Ten queries used in the experiments are defined as follows:

1. Planning a thesis/dissertation on a whiteboard with my PhD student, who was wearing a blue and black stripey top... in my office in 2016. We were using blue, black and green pens. After this, I went back to work at my computer. It was on the 27th of September.
2. I was organizing technology devices (phones, ipads, etc) on the wooden floor at home in an attempt to show a lifeloggers toolkit. There was a phone, an ipad, an ipad mini, a book, and other devices on a Sunday evening in 2016.
3. I was taking a photo of a lake with a DSLR camera. It was my Sony camera. I was driving outside of Sheffield before and after stopping at the lake. It was in 2015 on a Saturday.
4. I was taking a photo of grandfather clocks while shopping in the UK. It was a Saturday in an antique store in March 2015. I had driven a rental car to the store.
5. I was going into Northside Shopping Centre. I was there to get new keys. I drove to the shopping center from work and then I drove home. It was in 2015 in the morning time.
6. Drinking a bottle of Budweiser beer at home. This was during a BBQ in the evening in the summer of 2018. I had driven back home in someone else’s car before putting on the BBQ and getting the beer on a dull evening.
7. I was lost and looking for directions on a street, close to an Asian restaurant called Maple Leaf. It was in the late afternoon or evening and it was in Wexford. I had driven there in 2015.
8. Colleague in my office; she was carrying a large paper envelope full of documents. The envelope looked very heavy. She was wearing red trousers, a white shirt, and a polka-dot top. I remember my office door was open. It was in September in 2016. On the 27th I think, in the afternoon.
9. Eating a large plate of scrambled egg at home, alone in the late afternoon. I was in my living room, with the TV on and using my phone. I was sitting on my red chair with a green exercise mat visible. It was in 2016.
10. Birds in a cage, a yellow one on the lower left. There was also one box with a small, GREEN old car (Beetle-like). No, the car was BLUE! It was in 2018 in May. I think it was a Sunday.

## Appendix B: Location Categories

**Table 3:** Location categories

ID	Name	ID	Name
1	Airport	17	Home
2	Antique store	18	Hotel
3	Apartment	19	Howth
4	Bank	20	Office
5	Bar, pub	21	Park
6	Bus stop	22	Pharmacy
7	Car	23	Plane
8	Castle	24	Restaurant
9	Church	25	Shop
10	Coffee shop	26	Shopping Center
11	Convenience store	27	Sister home
12	DCU	28	Station
13	Dental clinic	29	Store
14	Department store	30	Street
15	Embassy	31	University
16	Hall	32	Unknown

## Footnotes

1 https://www.elastic.co

2 https://cloud.google.com/vision/docs/ocr

3 https://azure.microsoft.com/en-us/services/cognitive-services/computer-vision

4 https://www.djangoproject.com

5 https://reactjs.org

6 https://developers.google.com/maps/documentation/geocoding

7 https://www.nltk.org

8 https://www.elastic.co/guide/en/elasticsearch/reference/current/query-dsl.html

9 https://www.elastic.co/guide/en/elasticsearch/reference/current/query-dsl-query-string-query.html

10 https://www.elastic.co/guide/en/elasticsearch/reference/current/query-dsl-term-query.html

11 An evaluation server used in LSC’21 for evaluating retrieval systems. It is available at https://github.com/dres-dev/DRES
